# Isolated Gracilis Muscle Tear in a Ballet Dancer: A Case Report

**DOI:** 10.7759/cureus.117985

**Published:** 2026-10-15

**Authors:** Wei-Ting Wu, Yu-Chun Hsu, Ke-Vin Chang, Levent Özçakar

**Affiliations:** 1 Physical Medicine and Rehabilitation, National Taiwan University Hospital Bei-Hu Branch, Taipei, TWN; 2 Physical Medicine and Rehabilitation, Hacettepe University Medical School, Ankara, TUR

**Keywords:** gracilis muscle, rehabilitation, tear, thigh, ultrasonography

## Abstract

Isolated gracilis muscle tears are uncommon and may be overlooked because their clinical presentation can mimic injuries of the adductor or hamstring muscles. We report a 33-year-old female ballet dancer who developed acute posteromedial thigh pain while rising from a split position. Symptoms persisted despite initial conservative treatment, and she presented three weeks after the injury. Physical examination revealed tenderness along the medial thigh, with mild pain during resisted knee flexion, hip adduction, and internal rotation. Musculoskeletal ultrasonography demonstrated a focal partial tear of the gracilis at the musculotendinous junction, characterized by a hypoechoic defect, disruption of the normal muscle architecture, and increased vascularity on color Doppler imaging. Conservative management consisting of activity modification with avoidance of strenuous exercise and low-level laser therapy was initiated, with the visual analog scale (VAS) score decreasing from 7 to 0 within two weeks. At the two-month follow-up, the patient remained asymptomatic and had begun graded exercises in preparation for returning to ballet training. This report suggests that isolated gracilis injury should be considered in the differential diagnosis of acute posteromedial thigh pain after a mechanism involving rapid lengthening and active contraction. It illustrates how targeted musculoskeletal ultrasonography may assist diagnosis and follow-up when an isolated gracilis injury is clinically suspected.

## Introduction

Thigh muscle strains are common in athletes; however, isolated gracilis muscle injuries have been infrequently reported in the literature [1,2]. Their true incidence remains unclear, and they may be underrecognized because their clinical presentation can overlap with injuries of the adductor or hamstring muscles. The gracilis is a superficial, strap-like muscle that spans both the hip and knee joints and functions primarily in hip adduction and knee flexion while also contributing to medial rotation of the tibia. Because the gracilis spans both the hip and knee joints, it can be substantially lengthened across both joints during movements involving marked hip abduction or flexion with knee extension [1].

As the clinical presentation may overlap with that of hamstring or adductor injuries and other causes of posteromedial thigh pain, isolated gracilis injury may be overlooked. Musculoskeletal ultrasonography can provide direct visualization of the gracilis and surrounding structures [3,4]. We report the case of an isolated gracilis musculotendinous tear in a ballet dancer, describing the injury mechanism, ultrasonographic findings, and short-term clinical follow-up.

## Case presentation

A 33-year-old female ballet dancer presented with a three-week history of left posteromedial thigh pain. The injury occurred while she was practicing the splits. As she attempted to rise from the split position, she heard an audible “pop,” followed immediately by severe pain extending from the posteromedial thigh to the posteromedial aspect of the knee. The pain was initially severe enough to impair ambulation. She initially received topical medication and oral nonsteroidal anti-inflammatory drugs; however, her symptoms persisted. Transcutaneous electrical nerve stimulation was subsequently attempted but aggravated the pain. Because of persistent symptoms, she presented to our clinic.

On physical examination, tenderness was present along the medial thigh, extending from the inguinal region to the posteromedial aspect of the knee, without obvious swelling or ecchymosis. Her pain intensity was rated as 7 on the visual analog scale (VAS) [5]. Active and passive straight-leg raising with the knee extended did not reproduce her symptoms. Mild pain was elicited during active and resisted knee flexion. Hip adduction and internal rotation mildly reproduced the medial thigh pain, whereas hip abduction was painless. No additional abnormalities were identified on examination of the lumbar spine and hip joint.

Musculoskeletal ultrasound examination was performed using an Aplio 300 ultrasound system equipped with a 14L5 linear-array transducer (5-14 MHz; Canon Medical Systems Corp., Otawara, JPN). For color Doppler imaging, the Doppler frequency was set at 5 MHz and the color gain at 41. The adductor magnus, semitendinosus, and semimembranosus muscles and tendons appeared intact. The distal pes anserinus and pes anserine bursa were unremarkable, as was the posterior horn of the medial meniscus.

Further scanning along the superficial structures of the medial thigh revealed a focal partial tear of the gracilis at the musculotendinous junction. Short-axis ultrasonography demonstrated a focal hypoechoic defect with partial disruption of the normal muscle architecture. Long-axis color Doppler imaging demonstrated increased vascularity around the lesion (Figure 1). The defect measured approximately 14.51 × 9.59 × 7.50 mm (length × width × depth) and was located approximately 7.74 mm beneath the skin surface. The contralateral gracilis showed no corresponding sonographic abnormality. A small associated hematoma was also observed, whereas aspiration was not considered necessary. Dynamic assessment during muscle contraction was not performed because of pain. The sonographic appearance was compatible with a partial gracilis musculotendinous tear. Using the British Athletics Muscle Injury Classification as a descriptive framework, the lesion was considered comparable to a grade 2b injury; however, this classification was originally developed for MRI assessment [6].

**Figure 1:**
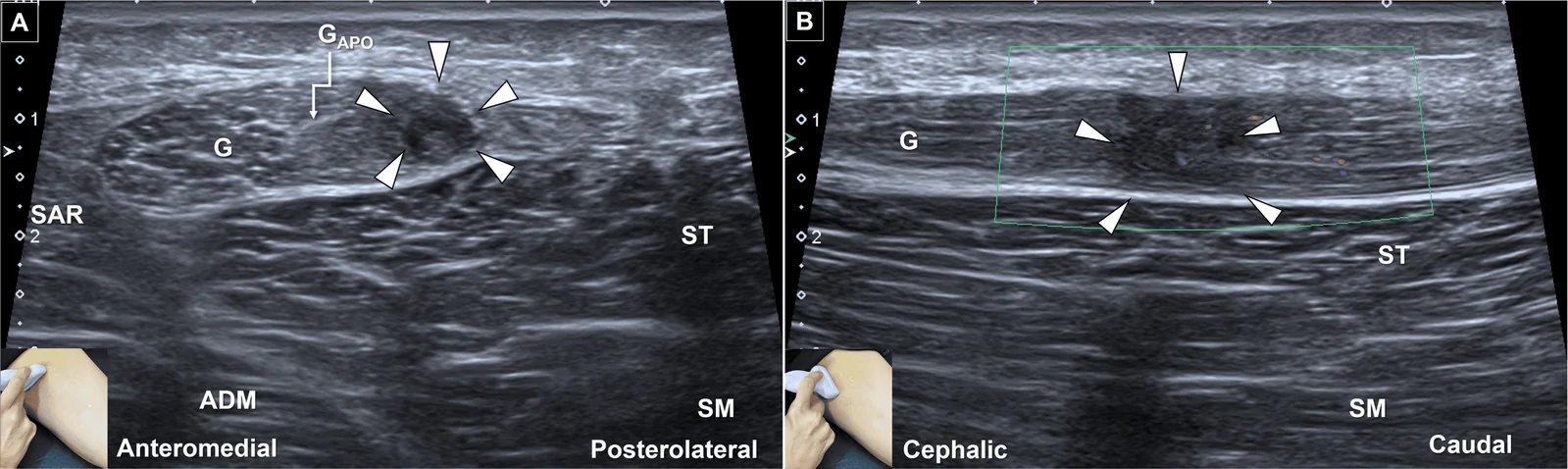
Ultrasound findings of the gracilis musculotendinous tear Short-axis (A) and long-axis color Doppler (B) ultrasound images demonstrate a partial tear of the gracilis at the musculotendinous junction, characterized by a focal hypoechoic defect and disruption of the normal muscle architecture. Increased vascularity is observed at the injury site on color Doppler imaging (B). White arrowheads delineate the injured region. G: Gracilis muscle; GAPO: Gracilis aponeurosis; SM: Semimembranosus muscle; ST: Semitendinosus muscle; ADM: Adductor magnus; SAR: Sartorius

The patient was advised to temporarily discontinue sports training and stretching exercises, and low-level laser therapy was applied to the injured region. Following these conservative measures, her pain improved, with the VAS score decreasing from 7 to 0 within two weeks. Follow-up ultrasonographic examinations demonstrated progressive resolution of the focal gracilis abnormality with partial restoration of the normal muscle architecture (Figure 2). At the two-month follow-up, she remained completely asymptomatic and had begun graded exercises in preparation for returning to ballet training but had not yet resumed ballet.

**Figure 2:**
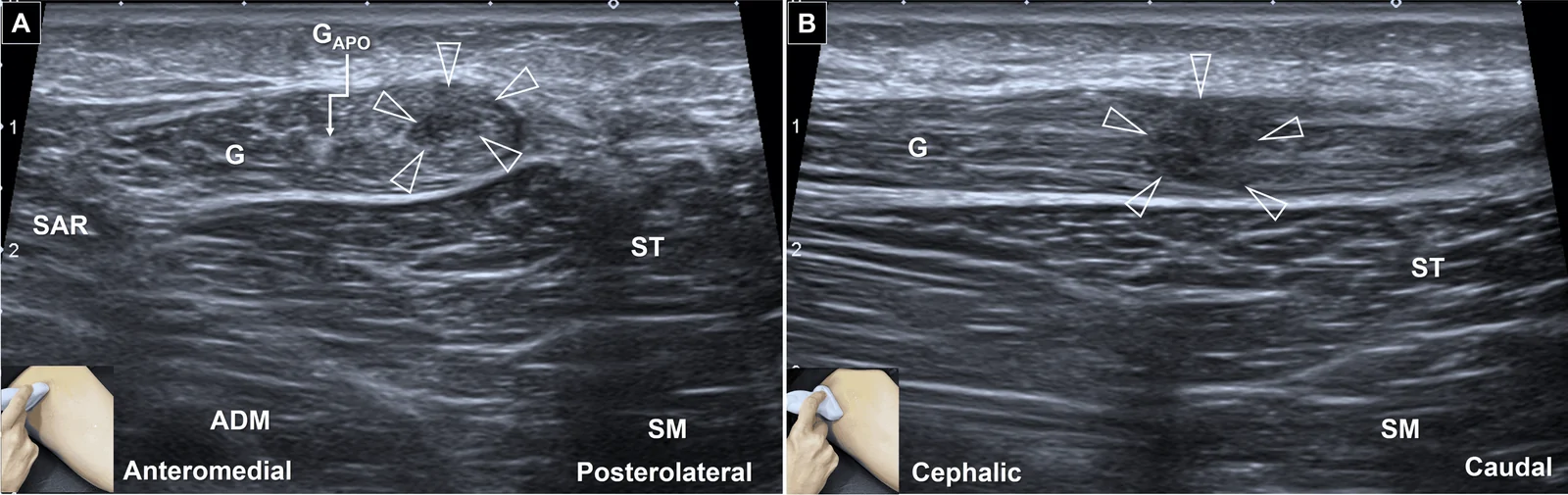
Follow-up ultrasound findings of the gracilis musculotendinous tear Short-axis (A) and long-axis (B) ultrasound images demonstrate progressive resolution of the focal gracilis abnormality with partial restoration of the normal muscle architecture. Open arrowheads indicate the residual hypoechoic area with ongoing architectural restoration. G: Gracilis muscle; GAPO: Gracilis aponeurosis; SM: Semimembranosus muscle; ST: Semitendinosus muscle; ADM: Adductor magnus; SAR: Sartorius

## Discussion

The gracilis is a superficial, strap-like muscle of the medial compartment of the thigh and is commonly classified as part of the hip adductor group. It originates from the inferior pubic ramus and descends along the medial thigh before continuing as a distal tendon that inserts on the proximal anteromedial tibia as part of the pes anserinus, together with the sartorius and semitendinosus tendons. As a biarticular muscle, the gracilis contributes to hip adduction, knee flexion, and medial rotation of the tibia [7].

Isolated gracilis muscle tears are uncommon and may be underrecognized because medial or posteromedial thigh pain is more frequently attributed to injuries involving the adductor or hamstring muscle groups [8,9]. Pedret et al. [1] reported seven athletes with isolated gracilis injuries, including three professional dancers, with different injury mechanisms described among the cases. Notably, one of the professional dancers sustained the injury while performing the splits with the affected hip flexed. All lesions in their series involved the gracilis myotendinous region.

In our case, the injury occurred as the patient attempted to rise from a split position, accompanied by an audible 'pop' and immediate severe posteromedial thigh pain. During a split maneuver, the gracilis is substantially lengthened across both the hip and knee joints. Transitioning from this position likely exposed the gracilis musculotendinous unit to high tensile load while it was lengthened across both joints; however, the precise contraction type could not be determined retrospectively. Clinical diagnosis may be challenging because the gracilis lies anatomically between the adductor and hamstring muscle groups, and its symptoms may mimic more common causes of medial thigh or posteromedial knee pain. Differential diagnoses include injuries of the adductor muscles [10], semitendinosus [11], and semimembranosus [12]; pes anserine pathology; and referred pain from the hip or lumbar spine. In our patient, no additional abnormalities were identified on examination of the lumbar spine and hip joint, while ultrasonography showed no abnormalities in the evaluated adjacent muscles and pes anserine structures, supporting the clinical diagnosis of an isolated gracilis injury.

Musculoskeletal ultrasonography played an important role in establishing the diagnosis in this case. In ambulatory musculoskeletal practice, timely access to appropriate imaging may facilitate the diagnostic pathway for selected presentations by allowing structural abnormalities to be evaluated in conjunction with the clinical examination [13,14]. Despite the extensive distribution of tenderness along the medial thigh, the physical examination findings were relatively nonspecific, with negative straight-leg raising and only mild symptom reproduction during provocative maneuvers. Targeted ultrasonography localized the lesion to the gracilis musculotendinous junction, demonstrating focal disruption of the normal muscle architecture and increased vascularity on color Doppler imaging. Simultaneous examination of the adjacent medial hamstrings and pes anserine structures revealed no abnormalities, further supporting an isolated gracilis injury. Chen et al. [2] also reported a distal gracilis myotendinous tear initially identified by point-of-care ultrasonography and subsequently confirmed by MRI. Taken together, these observations support the use of targeted ultrasonography for identifying a gracilis lesion corresponding to the clinical presentation and assessing adjacent structures. Clinical correlation remains essential, and MRI may be considered when ultrasonographic findings are equivocal or when a more extensive injury pattern is suspected.

Published reports of isolated gracilis injuries have generally described non-operative management [1,2]; however, evidence defining optimal rehabilitation for gracilis tears remains limited, and rehabilitation principles are often extrapolated from adductor and hamstring injury management [15]. Such approaches typically involve progressive strengthening and graded loading, followed by gradual progression to sport-specific activities [8]. Within such rehabilitation approaches, limiting excessive stretching during the early phase may be considered to avoid high tensile loading of the healing musculotendinous unit, although this recommendation is extrapolated from related muscle-injury rehabilitation rather than gracilis-specific evidence. In the series by Pedret et al. [1], all seven athletes were treated nonoperatively and returned to full athletic performance within six weeks, with a mean return-to-sport interval of 35.6 days.

Ultrasonography demonstrated progressive healing of the gracilis lesion, paralleling the rapid clinical improvement. However, return-to-sport decisions should integrate clinical and functional recovery rather than rely solely on morphological restoration on follow-up ultrasonography. At the two-month follow-up, the patient remained asymptomatic and began a gradual return to ballet training. Although the contribution of low-level laser therapy or any individual component of the conservative management cannot be determined from a single case, the clinical and sonographic recovery supports the favorable prognosis of conservatively managed partial gracilis tears.

## Conclusions

Isolated gracilis musculotendinous injury should be considered in athletes with acute medial or posteromedial thigh pain after a high-tensile movement, including transitions from split positions. When performed by an experienced examiner, targeted musculoskeletal ultrasonography may localize a suspected gracilis lesion, assess accessible adjacent structures, and document interval structural changes during follow-up. Ultrasonographic findings should be interpreted in conjunction with the clinical presentation, and MRI remains useful when ultrasound findings are equivocal or a broader injury pattern is suspected. Clinical and functional recovery should remain central to return-to-sport decisions.

## References

[1] Pedret C, Balius R, Barceló P (2011). Isolated tears of the gracilis muscle. Am J Sports Med.

[2] Chen YT, Mills C, von Rickenbach KJ, McInnis KC (2022). Distal gracilis tear in an equestrian. Am J Phys Med Rehabil.

[3] Chang KV, Wu WT, Özçakar L (2019). Ultrasound imaging and rehabilitation of muscle disorders: part 1 traumatic injuries. Am J Phys Med Rehabil.

[4] Kara M, Ata AM, Kaymak B, Özçakar L (2020). Ultrasound imaging and rehabilitation of muscle disorders: part 2: nontraumatic conditions. Am J Phys Med Rehabil.

[5] Huskisson EC (1974). Measurement of pain. Lancet.

[6] Pollock N, James SL, Lee JC, Chakraverty R (2014). British athletics muscle injury classification: a new grading system. Br J Sports Med.

[7] Możdżeń K, Fibiger G, Malczak M (2026). Morphometry and functional topography of the pes anserinus complex: a standardized cadaveric study. Ann Anat.

[8] Heiderscheit BC, Sherry MA, Silder A, Chumanov ES, Thelen DG (2010). Hamstring strain injuries: recommendations for diagnosis, rehabilitation, and injury prevention. J Orthop Sports Phys Ther.

[9] Malliaropoulos NG (2012). Non contact Hamstring injuries in sports. Muscles Ligaments Tendons J.

[10] Hsu CY, Wu WT, Hsu YC, Chang KV, Özçakar L (2026). Ultrasonographic diagnosis of an ischiocondylar adductor magnus partial tear: an uncommon cause of ischial pain. Diagnostics.

[11] Lin TY, Chang KV, Wu WT (2026). USMSIT/NMUSIT fundamental guide on diagnostic and interventional hip ultrasonography. J Med Ultrasound.

[12] Wu WT, Mezian K, Ricci V, Chang KV, Ozcakar L (2023). Semimembranosus muscle tear masquerading as a hypervascular tumor: ultrasonographic differential diagnosis for gluteal pain syndrome. Med Ultrason.

[13] Chow CL, Shum JS, Hui KT, Lin AF, Chu EC (2023). Optimizing primary healthcare in Hong Kong: strategies for the successful integration of radiology services. Cureus.

[14] Lee WT, Chu EC, Chau C (2023). Understanding the utilization, challenges, and attitudes towards laboratory testing and radiological imaging among chiropractors in Hong Kong: a cross-sectional survey. Cureus.

[15] Serner A, Weir A, Tol JL, Thorborg K, Lanzinger S, Otten R, Hölmich P (2020). Return to sport after criteria-based rehabilitation of acute adductor injuries in male athletes: a prospective cohort study. Orthop J Sports Med.

